# An Indoor Pedestrian Positioning Method Using HMM with a Fuzzy Pattern Recognition Algorithm in a WLAN Fingerprint System

**DOI:** 10.3390/s16091447

**Published:** 2016-09-08

**Authors:** Yepeng Ni, Jianbo Liu, Shan Liu, Yaxin Bai

**Affiliations:** 1 Computer and Network Center, Communication University of China, No. 1 Dingfuzhuang East Street, Chaoyang District, Beijing 100024, China; 2 Information Engineering Institute, Science and Technology Department, Communication University of China, No. 1 Dingfuzhuang East Street, Chaoyang District, Beijing 100024, China; ljb@cuc.edu.cn (J.L.); liushan@cuc.edu.cn (S.L.); 3 School of Journalism, Faculty of Journalism and Communication, Communication University of China, No. 1 Dingfuzhuang East Street, Chaoyang District, Beijing 100024, China; rhbyx@cuc.edu.cn

**Keywords:** pedestrian positioning, fuzzy pattern recognition algorithm, RSSI variation trend, hidden markov model, smartphone, fingerprint system

## Abstract

With the rapid development of smartphones and wireless networks, indoor location-based services have become more and more prevalent. Due to the sophisticated propagation of radio signals, the Received Signal Strength Indicator (RSSI) shows a significant variation during pedestrian walking, which introduces critical errors in deterministic indoor positioning. To solve this problem, we present a novel method to improve the indoor pedestrian positioning accuracy by embedding a fuzzy pattern recognition algorithm into a Hidden Markov Model. The fuzzy pattern recognition algorithm follows the rule that the RSSI fading has a positive correlation to the distance between the measuring point and the AP location even during a dynamic positioning measurement. Through this algorithm, we use the RSSI variation trend to replace the specific RSSI value to achieve a fuzzy positioning. The transition probability of the Hidden Markov Model is trained by the fuzzy pattern recognition algorithm with pedestrian trajectories. Using the Viterbi algorithm with the trained model, we can obtain a set of hidden location states. In our experiments, we demonstrate that, compared with the deterministic pattern matching algorithm, our method can greatly improve the positioning accuracy and shows robust environmental adaptability.

## 1. Introduction

Nowadays, with the rapid development of smartphones and wireless networks, people can conveniently access location-based services (LBSs) with mobile applications. Location information has become the cornerstone of several new technologies and applications including big data, cloud computing, Internet of things, and online-to-offline. The accurate positioning of the users is the key to providing LBS guaranteed by quality of experience (QoE).

The localization technology is mainly divided into outdoors and indoors. The outdoors localization technology is relatively mature, and mainly includes the Global Positioning System (GPS) and cellular positioning systems [1]. However, for indoor localization, GPS signals cannot reach the receivers and the positioning accuracy is too low to support indoor services. For this purpose, people have done a lot of research with different technologies, and developed RFID positioning systems, infrared ray positioning systems, Bluetooth positioning systems, ZigBee positioning systems, ultrasonic positioning systems, vision positioning systems, voice recognition positioning systems, and WLAN (Wireless Local Area Network) fingerprint positioning systems, etc. [2]. Among these, the WLAN fingerprint positioning technology has become a research hotspot in the indoor localization field on account of the popularity of WLAN signals in indoor environments.

**Figure 1:**
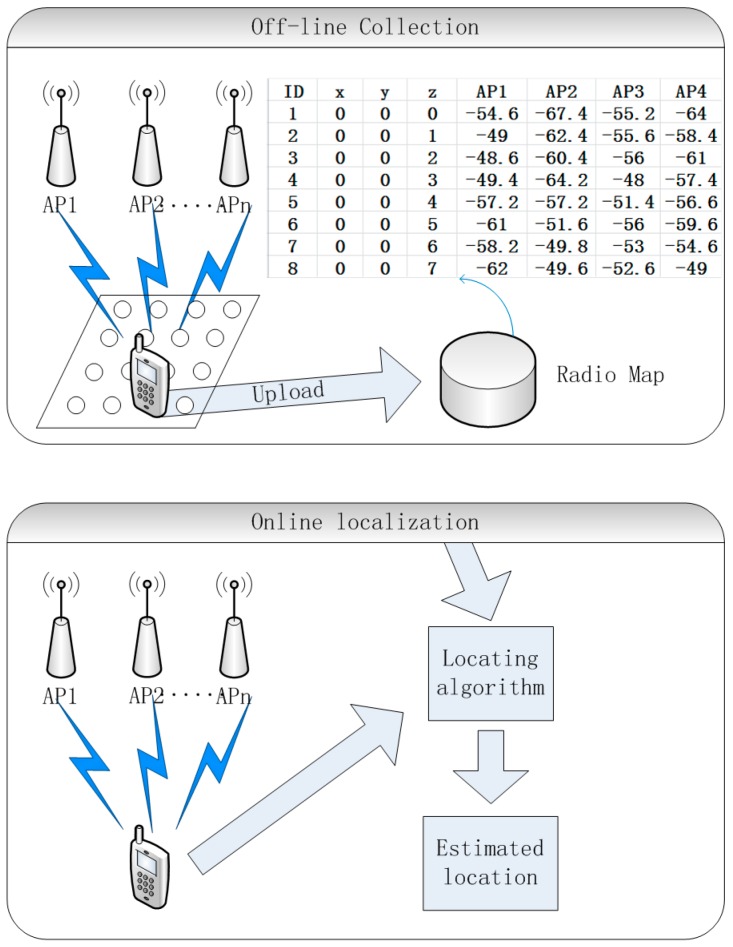
The workflow of a WLAN (Wireless Local Area Network) fingerprint system.

The WLAN fingerprint system is an economical and practical positioning system. Bahl et al. [3,4] have introduced the basic principle of WLAN fingerprinting. The system workflow is shown in Figure 1. In the off-line phase, users sequentially record Received Signal Strength Indicator (RSSI) values with current position coordinates into a database; this process is often called calibration and the database is called a radio map. In the on-line localization phase, a user enters into a WLAN environment with terminals and samples the RSSI. The user position coordinates are estimated by comparing the RSSI sample against the radio map to determine the closest signal distance.

Although there are some classical WLAN fingerprint systems, the research objective is always focused on a static target. There are few papers discussing pedestrian positioning, which is more relevant in a real environment. Due to distance variation and human body interference, the RSSI of moving targets will fluctuate over a large range, especially at a relatively slow speed [5]. Moreover, the sampling time is limited, which means we cannot use the averaging method to reduce the influence of noise because of the too small sample size. The above factors lead to severe distortions in the WLAN fingerprint systems that sample RSSI, thus seriously compromising the positioning accuracy.

In order to offer users LBS services with better positioning accuracy in a real environment, in this paper, a pedestrian positioning method using a Hidden Markov Model (HMM) [6] with a Fuzzy Pattern Recognition (FPR) algorithm in a WLAN fingerprint system is proposed. Firstly, we use the FPR algorithm to rebuild the pre-existing radio map; then, we utilize a forward-backward algorithm to train the HMM using the planned path and the rebuilt radio map; finally, we can utilize a Viterbi algorithm to estimate the pedestrian position using the constructed HMM. This method take full advantage of the prior information of the WLAN fingerprint system and user information (RSSI, user trajectories), and our experiments indicate that this method can effectively improve the positioning accuracy, consequently improving the user experience.

This paper is organized as follows: Section 2 briefly introduces the related work. Section 3 proposes a fuzzy pattern recognition algorithm to improve pedestrian positioning accuracy. Section 4 elaborates on how to train the HMM with the FPR algorithm in the fingerprint system, and use the Viterbi algorithm to estimate pedestrian position. Section 5 describes the experimental testbed and conducts the experiment before the experimental results are analyzed and compared. The last section concludes the research contents in this paper and gives some suggestions of future research.

## 2. Related Work

In recent years, research on indoor fingerprint positioning has been conducted actively. We will discuss some representative works here, especially the ones that employ HMM in their systems. To reduce the calibration work, Wallbaum et al. [7,8] employed a radio propagation model and HMM to quickly implement an indoor positioning system. Given some independent assumptions, it uses a discrete probability distribution to describe all hypothetical positions, and this probability distribution is only updated when new RSSI data is collected or the user has moved. The position is estimated by weighing the different hypotheses. This method needs high computation overhead on user terminals, and the accuracy is not good due to the fact the radio propagation model can hardly model realistic environments.

Seitz et al. [9] improve upon the updating method mentioned by Wallbaum et al. [8], and consider the angle factor in the movement updating procedure. Using this method, the proposed HMM directly includes the movement measurements. Although this method could achieve a relatively better positioning accuracy, the experiments were carried out in a clean environment such as a market with no visitors and it was assumed that the fluctuating RSSI followed a Gaussian distribution. It did not consider that there is a mismatch between RSSI measurements in movement and the fingerprint database.

Liu et al. [10] presented a lightweight and robust indoor positioning system. To reduce the calibration work, this system employs a Weibull distribution to represent the distribution of the RSSI over time. Then, it uses an accelerometer sensor to provide the information on the movement distance to calculate the transition probability of the HMM, which will reduce the computational complexity of the positioning process. However, the Weibull distribution cannot accommodate the fluctuating RSSI samples, and the transition probability hardly reflects the real state transition process.

A novel solution named HIPE (Hybrid Indoor Positioning Engine) [11] has applied data fusion to indoor positioning. The HIPE is an engine which fuses the motion dynamics information of a mobile user with the RSSI. Two algorithms are employed in the HIPE, and which one is selected depends on the need for real-time service. The problem with this solution is that the HIPE must install some software on the user’s smartphones and this relies on the computational resources of the smartphone. Besides that, the authors do not outline an explicit method to determine the transition probability.

Coleri et al. [12] employed a HMM-based positioning algorithm to position vehicles in constrained routes by exploiting GSM RSSI data. Compared with the previously proposed HMM techniques, their algorithm trains HMM using the driver behavior statistics for the road segments instead of employing the driver behavior as an equal-probability distribution for each of the adjacent reference points (RPs) without any training. They do not take into account that the speed of each movement is different, but rather assume the vehicle is moving with uniform speed on the road segments.

## 3. Fuzzy Pattern Recognition Algorithm

In previous studies, there is a simple scheme of pattern recognition in indoor positioning. It is assumed that each position is associated with a unique set of RSSI values, so the distance between a user’s measured location and the reference location can be calculated by Equation (1):

(1) $$d\left(s,r\right)=\sqrt{\overset{n}{\underset{i\;=\;1}{\sum }}{\left(s_{i}-r_{i}\right)}^{2}}$$

where $s=\left[s_{1}\cdots s_{i}\right]$ and $r=\left[r_{1}\cdots r_{i}\right]$ represent the RSSI vector, respectively, n represents the number of Access Points (APs), d(s,r) represents the Euclidean distance between two locations. The user coordinates are computed by the Euclidean distance from the measured location to all reference locations stored in the radio map, and the RSSI vector with the smallest distance is the nearest location to the true user location. This method is called the Nearest Neighbor (NN) algorithm, and an enhanced method employing the average K nearest RP is called KNN. Both are widely known deterministic pattern matching algorithms.

**Figure 2:**
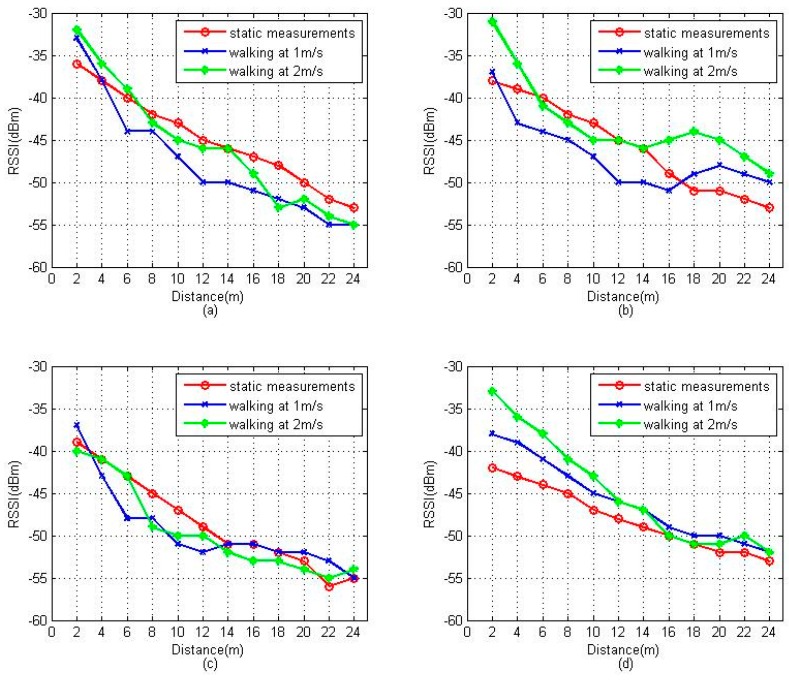
RSSI variation trend at different walking speed with different devices: (**a**) Samsung S5 with TP-LINK TL-WR840N; (**b**) XIAOMI, (XIAOMI, Beijing, China), HM2 with TP-LINK TL-WR840N; (**c**) Samsung S5 with MERCURY MW325R; (**d**) XIAOMI HM2 with MERCURY MW325R.

Although this deterministic pattern matching algorithm with better positioning accuracy can be easily implemented in a WLAN fingerprint system, it is based on an accurate radio map and high precision sampling of RSSI, the latter being almost impossible while people are walking due to the vulnerabilities of the RSSI. Figure 2 shows the fluctuation of RSSI sampling by a user carrying a smartphone walking at different speeds within radio range. Here, we assume that the maximum walking speed is no more than 2 m per second (2 m/s), as mentioned by Alexander et al. [13]. To show that the RSSI variation trend is independent of the device used, we repeated the experiment several times with two APs and two smartphones, both from different vendors. For comparison, the static positioning measurements of RSSI, which are usually stored in the radio map, are also given in the figure. Pu et al. [14] have presented similar results, but collected at a much faster speed.

Compared with the static positioning measurements, we can see that the RSSI values show significant variations while people are walking due to the sophisticated propagation of the radio signal. The RSSI deviations caused by the different measurement conditions (static or dynamic) will introduce critical positioning errors. Meanwhile, we find that most of the dynamic positioning measurements also follow the propagation and attenuation law, whereby the RSSI fades with the increasing distance between the measuring point and the AP. From this rule, the RSSI variation trend can be used to indicate the relative direction and distance from the initial location to the positioning point. For example, if the RSSI is stronger than at the initial location, it means the positioning point is closer to the AP than the initial location. If there are enough RPs in the area, it can be easily compared to determine the relative locomotion of the pedestrian.

**Figure 3:**
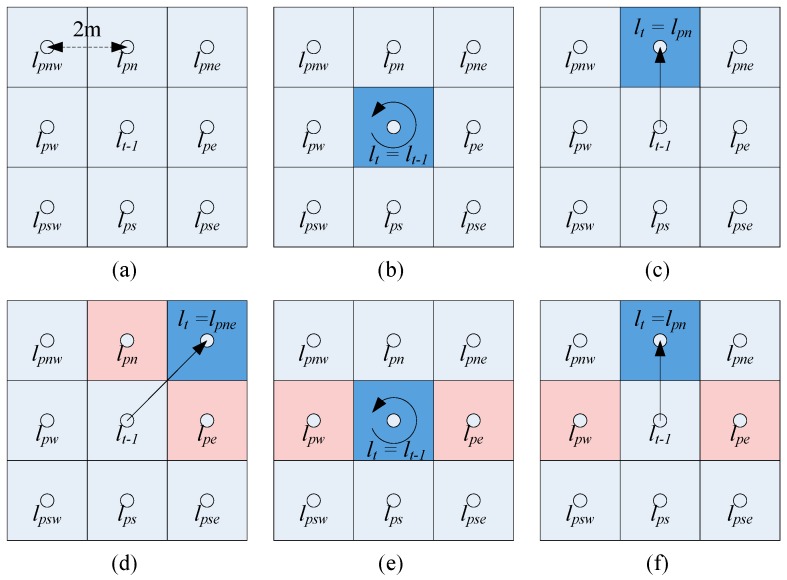
Schematic of the fuzzy pattern recognition algorithm: (**a**) nine RPs in a grid area and the interval of the RPs is 2 m; (**b**) $l_{t}=l_{t\;-\;1}$ when the RSSI has no variation or a similar variation in four directions; (**c**) $l_{t}=l_{pn}$ when the RSSI variation trend follows the trajectory from $P_{0}$ to $P_{n}$; (**d**) $l_{t}=l_{pn}$ when the RSSI has a similar variation trend in the north and east, we assume the RSSI variations follow the trajectory from $P_{0}$ to $P_{\text{ne}}$; (**e**) $l_{t}=l_{t\;-\;1}$ when the RSSI has a similar variation in the east and west; (**f**) $l_{t}=l_{pn}$ when the RSSI has a similar variation in the east, west and north, we assume the RSSI variations follow the trajectory from $P_{0}$ to $P_{n}$.

Based on above analysis, we propose a fuzzy pattern recognition algorithm to estimate the pedestrian position, thus reducing the accuracy loss caused by people walking. It uses the RSSI variation trend to replace the actual RSSI value, and the HMM observation state is also indicated by the RSSI variation trend. Relative to the last observation state, RSSI has three main variation trends in HMM, namely, stronger, weaker and flat. To simplify our algorithm, we use the numbers 1, −1, and 0 to respectively represent these three trends. Except for the initial observation state, all the subsequent observation states are indicated by three numbers. For example $O_{\left(t,t\;-\;1\right)}=\left(-1,\;0,\;\cdots 0,\;0,\;1,\;1,\;-1\right)$, presents the variation of RSSI value at time t compared to time (t−1) in a localization area that contains n APs. The fuzzy pattern recognition arithmetic steps are as follows:

(1) Given an initial RP $P_{0}$ in the grid area established via static positioning or user feedback, the RSSI vectors and the position coordinates of $P_{0}$ are respectively $s_{t\;-\;1}=\left({\text{rss}}_{1\left(t\;-\;1\right)},{\;\text{rss}}_{2\left(t\;-\;1\right)},\;\cdots {\text{rss}}_{n\left(t\;-\;1\right)}\right)$ and $l_{t\;-\;1}=\left(x_{p0},\;y_{p0}\right)$.
(2) Measure RSSI vectors $s_{t}=\left({\text{rss}}_{1t},{\;\text{rss}}_{2t},\;\cdots {\text{rss}}_{\text{nt}}\right)$ during pedestrian walking per second, and compare each AP’s RSSI of $s_{t}$ with $s_{t\;-\;1}$. Considering the signal random disturbance, we give a ±1 dB m threshold to compensate for the influence of RSSI fluctuation. $O_{\left(t,t\;-\;1\right)}$ is the comparison result. The pseudo-code of this procedure is shown as follows:
  there are:
  s_t_ = (rss_1t_, rss_2t_,…rss_nt_);
  s_t – 1_=(rss_1(t–1)_,rss_1(t–1)_,…rss_1(t–1)_)
  $O_{\left(t,t\;-\;1\right)}=\left(o_{1},{\;o}_{2},\;\cdots o_{n}\right)$;
  for(j = 1; j ≤ n; j++)
  {
  if (${\text{rss}}_{\text{jt}}$ > $\left({\text{rss}}_{j\left(t\;-\;1\right)}+1\right)$)
  o_j_ = 1
  else if (${\text{rss}}_{\text{jt}}$ < $\left({\text{rss}}_{j\left(t\;-\;1\right)}-1\right)$)
  o_j_ = −1
  Else
  o_j_ = 0
  }
(3) Compare each AP’s RSSI of $P_{0}$ with the adjacent RPs in four directions, namely $P_{e}$, $P_{w}$, $P_{s}$ and $P_{n}$. The position coordinates of the four RPs are $l_{p_{e}}$, $l_{p_{w}},\;l_{p_{s}}$, $l_{p_{n}}$. By employing the above method, the comparison results are $O_{e},\;O_{w},\;O_{s}\;\text{and}\;O_{n}$.
(4) For estimating the position of the pedestrian, we need to separately compare the RSSI variation trend by performing a bitwise logical XOR operation between $O_{\left(t,t\;-\;1\right)}$ and $O_{e},\;O_{w},\;O_{s},O_{n}$. The results can determine the relative distance from the pedestrian to the four RPs, which can be used to deduce the approximate position of the pedestrian. The pseudo-code of this procedure is shown as follows:
  there are:
  $l_{t-1}$,$l_{p_{e}}$, $l_{p_{w}},\;l_{p_{s}}$, $l_{p_{n}},\;l_{p_{\text{ne}}},\;l_{p_{\text{se}}},\;l_{p_{\text{sw}}},\;l_{p_{\text{nw}}},l_{t}$;
  $O_{\left(t,t\;-\;1\right)}$, $O_{e},\;O_{w},\;O_{s},\;O_{n}$;
  $O_{e}\;⊕\;O_{\left(t,t\;-\;1\right)}=\left(x_{1e},x_{2e},\cdots x_{\text{ne}}\right)$;
  $O_{w}\;⊕\;O_{\left(t,t\;-\;1\right)}=\left(x_{1w},x_{2w},\cdots x_{\text{nw}}\right)$;
  $O_{s}\;⊕\;O_{\left(t,t\;-\;1\right)}=\left(x_{1s},x_{2s},\cdots x_{\text{ns}}\right)$;
  $O_{n}\;⊕\;O_{\left(t,t\;-\;1\right)}=\left(x_{1n},x_{2n},\cdots x_{\text{nn}}\right)$;
  $\left|O_{\left(t,t\;-\;1\right)}\right|$ = $\left|\left(o_{1},\;o_{2},\cdots o_{i}\right)\right|=\left(o_{1}^{\prime },\;o_{2}^{\prime },\cdots o_{i}^{\prime }\right)$;
  $O_{e}^{\prime }=\left(x_{1e}+x_{2e}+\cdots +x_{\text{ne}}\right)$;
  $O_{w}^{\prime }=\left(x_{1w}+x_{2w}+\cdots +x_{\text{nw}}\right)$;
$O_{s}^{\prime }=\left(x_{1s}+x_{2s}+\cdots +x_{\text{ns}}\right)$;
$O_{n}^{\prime }=\left(x_{1n}+x_{2n}+\cdots +x_{\text{nn}}\right)$;
$O_{\left(t,t\;-\;1\right)}^{\prime }=\left(o_{1}^{\prime }+o_{2}^{\prime }+\cdots +o_{n}^{\prime }\right)$;
if ($O_{\left(t,t\;-\;1\right)}^{\prime }=0\;\text{or}\;O_{\left(t,t\;-\;1\right)}^{\prime }\;\text{equal or approximately equal to all of}\;\left(O_{e}^{\prime },\;O_{w}^{\prime },\;O_{s}^{\prime },\;O_{n}^{\prime }\right)$)
$l_{t}=l_{t\;-\;1}$;
else if ($O_{\left(t,t\;-\;1\right)}^{\prime }\;\text{only equal or approximately equal to one of}\;\left(O_{e}^{\prime },\;O_{w}^{\prime },\;O_{s}^{\prime },\;O_{n}^{\prime }\right)$)
$l_{t}=\text{one of}\;\left(l_{p_{e}},\;l_{p_{w}},\;l_{p_{s}},\;l_{p_{n}}\right)$;
else if ($O_{\left(t,t\;-\;1\right)}^{\prime }\;\text{equal or approximately equal to two of}\;\left(O_{e}^{\prime },\;O_{w}^{\prime },\;O_{s}^{\prime },\;O_{n}^{\prime }\right)$)
{
if $(\text{two of}\;\left(O_{e}^{\prime },\;O_{w}^{\prime },\;O_{s}^{\prime },\;O_{n}^{\prime }\right)$ are adjacent )
$l_{t}=\text{one of}\;\left(l_{p_{\text{ne}}},\;l_{p_{\text{es}}},\;l_{p_{\text{sw}}},\;l_{p_{\text{wn}}}\right)$;
else
$l_{t}=l_{t\;-\;1}$;
}
else if ($O_{\left(t,t\;-\;1\right)}^{\prime }\;\text{equal or approximately equal to three of}\;\left(O_{e}^{\prime },\;O_{w}^{\prime },\;O_{s}^{\prime },\;O_{n}^{\prime }\right)$)
$l_{t}=\text{one of}\;\left(l_{p_{e}},\;l_{p_{w}},\;l_{p_{s}},\;l_{p_{n}}\right)$;

The “equal“ means all elements in the two vectors are totally equal, and the “approximately equal” means the two vectors have the largest number of identical elements in order. The $l_{p_{\text{ne}}},\;l_{p_{\text{es}}},\;l_{p_{\text{sw}}},\;l_{p_{\text{wn}}}$ respectively indicate the northeast, southeast, southwest and northwest RP coordinates ($P_{\text{ne}}$, $P_{\text{es}}$, $P_{\text{sw}}$, $P_{\text{wn}}$). A more visual description of this algorithm is shown as Figure 3.

Figure 3 contains all the possible cases of pedestrian movement if we consider the maximum walking speed is no more than 2 m/s, as mentioned above. To estimate the pedestrian position with this algorithm iteratively, we assume that the positioning result is RP $P_{\text{ne}}$ in the case of Figure 3d, although it is impossible for people to walk from $P_{0}$ to $P_{\text{ne}}$ at 2 m/s walking speed. From Figure 3 it is easy to see that the positioning accuracy of the fuzzy pattern recognition algorithm is limited by the interval between RPs. The comparison of positioning results under different measuring conditions (static or dynamic) will be discussed in the later experiments.

## 4. Pedestrian Positioning Method Based on HMM

As discussed in Section 2, some researchers employ HMM to implement moving target positioning, but all such approaches more or less have some defects and problems. To make up for the defects and solve the problem, we propose a novel scheme that combines the WLAN fingerprints system with the HMM. To decrease the accuracy loss caused by the vulnerability of WLAN signals, it uses a fuzzy pattern recognition method instead of the deterministic pattern matching algorithm in HMM. It uses the FPR algorithms to train HMM with user trajectories and estimate hidden location states through the Viterbi algorithm.

### 4.1. Mathematical Model of Pedestrian Positioning

**Figure 4:**
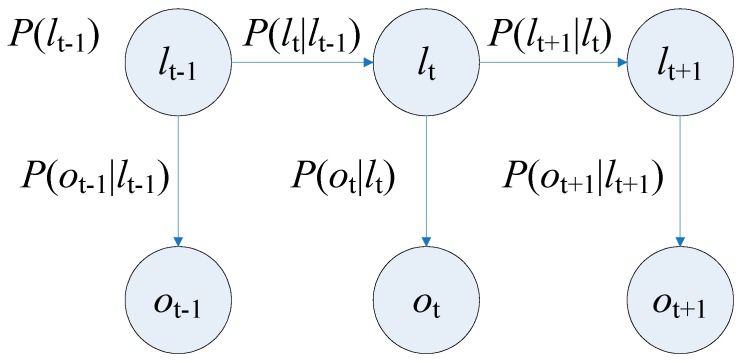
The architecture of the first-order HMM.

The Hidden Markov Model (HMM) is a well-known statistical model which has been successfully applied to acoustic treatment, biological information and natural language processing. Until now, it is still widely used in speech recognition [15]. Different from the traditional Markov model, HMM is a simple dynamic Bayesian network, where there are two kinds of states: hidden state and observation state. The hidden state transition process is a Markov process, and the observation states are generated by hidden Markov processes according to a certain probability distribution [16]. Figure 4 shows the basic architecture of a first-order HMM, where the term $l_{t}$ is the hidden state at time t, $o_{t}$ is the observation at time t, $P\left(l_{t}|l_{t\;-\;1}\right)$ is the transition probability from $l_{t\;-\;1}$ to $l_{t}$, $P\left(o_{t}|l_{t}\right)$ is the conditional probability when the observation is $o_{t}$ in the hidden state $l_{t}$.

Employing HMM can effectively improve pedestrian positioning accuracy in a WLAN fingerprint system. The HMM can be mathematically defined as a triple λ = {π,A,B} which includes three probability matrices, and two sets of states (L,O) in our pedestrian positioning method.

L is the location space, $L=\left\{l_{1},\;l_{2},\cdots ,l_{N}\right\}$, $l_{i},\left(1\leq i\leq N\right)$, each state represents a reference grid point with coordinates in the localization area [17], N is the total number of points.

V is the observation signal space, $V=\left\{v_{1},\;v_{2},\cdots ,v_{M}\right\}$, $v_{j},\left(1\leq j\leq M\right),$ each state represents a RSSI variation trend vector in a location state, M is the total number of possible vectors. The vector has three kinds of symbol, namely 1, 0, −1, which are respectively presented as $z_{-1}$, $z_{0}$, $z_{1}$.

π is an initial location state probability distribution, $\pi =P\left(l_{i}\right)\;\left(l_{i}\;\in \;L\right)$ is the probability of state $l_{i}$. Here, we assume that π follows a uniform probability distribution.

A is an N×N state transition matrix, $A=\left\{a_{\text{ij}}=P\left(L_{t\;+\;1}=l_{j}|L_{t}=l_{i}\right)\right\}\;\left(1\leq i,j\leq N\right)$, $a_{\text{ij}}$ is the probability of the transition from location $l_{i}$ to location $l_{j}$, thus there is $\sum_{j\;=\;1}^{N}a_{\text{ij}}=1\left(0\leq a_{\text{ij}}\leq 1\right)$. Because of the limitation of pedestrians’ walking speed (i.e., 2 m/s), if we divide the location area into 2 m ×2 m grids, we can consider that the transition probability is only valid between the adjacent grids, and the transition probability is zero in the case of cross grids. The transition probability of HMM is time invariant, so $P\left(L_{t}=l_{t}\right)$ solely depends on the last location state $l_{t\;-\;1}$.

B is a confusion matrix, $B=\left\{b_{i}\left(k\right)=P\left(v_{k}|l_{i}\right)\right\}\;\left(v_{k}\in O,l_{i}\in L\right)$, $b_{i}\left(k\right)$ is the emission probability when the RSSI variation trend is $v_{k}$ in location $l_{i}$ at time t, thus there is $\sum_{j\;=\;1}^{N}b_{i}\left(k\right)=1\left(0\leq b_{i}\left(k\right)\leq 1\right)$. Here, we assume that the emission probability solely depends on the location state at time t.

### 4.2. Pedestrian Positioning Using Viterbi Algorithm

**Figure 5:**
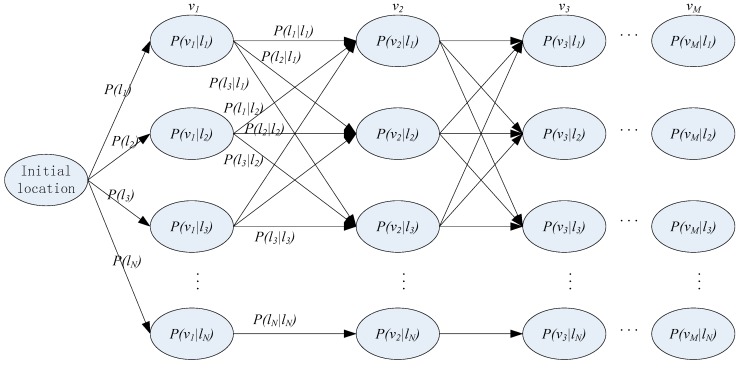
The trellis of the RSSI variation trends and the location transition.

Given a set of RSSI variation trends, $V=\left\{v_{1},\;v_{2},\cdots ,v_{M}\right\}$ and a settled HMM λ = {π,A,B}, the hidden location sequence $L=\left\{l_{1},\;l_{2},\cdots ,l_{N}\right\}$ can be estimated by employing the Viterbi algorithm. The Viterbi algorithm is proposed on the basis of dynamic programming for finding the optimal hidden state sequence [18]. The observed RSSI variation trends and the possible location transition are shown as a trellis in Figure 5.

To find the most likely sequence of the location, i.e., marking the path of the actual location transition in Figure 5, the Viterbi algorithm will be manipulated through four procedures as follows: Initialization, Recursion, Termination, and Path Backtracking [10]. Since there are a lot of resources describing the algorithm, it will not be covered here.

Through the procedures, we can learn that the transition probability has an important role in the Viterbi algorithm. Not only is the computation complexity of the Viterbi relative to the transition probability, but also the positioning accuracy is dependent on the transition probability. So far, the problem has been centered on how to calculate the transition probability.

### 4.3. Employing the FPR Algorithm to Estimate the Transition Probability

Transition probability is the most critical parameter in the pedestrian positioning model. The positioning accuracy of the Viterbi algorithm depends on the precision of the transition probability. To obtain the transition probability which can reflect the pedestrian walking pattern, we utilize the FPR algorithm to estimate the transition probability using a rebuilt radio map and planned path.

**Figure 6:**
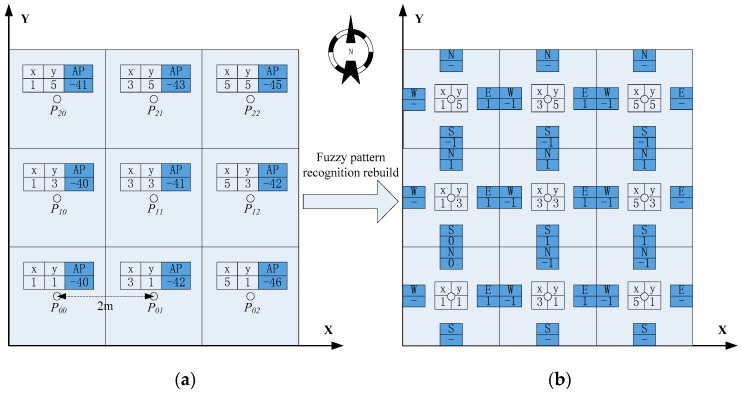
The comparison of two kinds of radio maps: (**a**) the pre-existing radio map; (**b**) the reconstructive radio map.

The pre-existing radio map stored the RSSI and the corresponding position coordinates. To reduce the RSSI deviation caused by different measurement conditions, we use the fuzzy pattern recognition algorithm to rebuild the pre-existing radio map. A comparison of the two kinds of radio maps is shown in Figure 6.

The left part of Figure 6 is a traditional radio map, where to keep things simple, we only give RSSI values of one AP in the radio map. The right part is the new radio map which is rebuilt by comparing the RSSI variation trend between RP and its adjacent RPs in four directions. Correspondingly, the HMM observation signal space also changes from the RSSI value to the RSSI variation trend.

In indoor areas, people will follow certain rules in moving, such as a limited walking speed, that there is no way through the walls, and that they walk in a straight line in a corridor. Using these rules, we can reasonably plan the user’s path, and use these trajectories as the HMM training samples so as to effectively improve the positioning accuracy. For training the HMM with the FPR algorithm, a set of consecutive RSSIs are recorded by volunteer sampling along the planned path. The planned path should contain all the RPs [17], so it will be able to obtain higher positioning accuracy. Because the FPR algorithm is sensitive to the direction of pedestrian travel, multiple training trajectories are provided in all four directions, as well.

**Figure 7:**
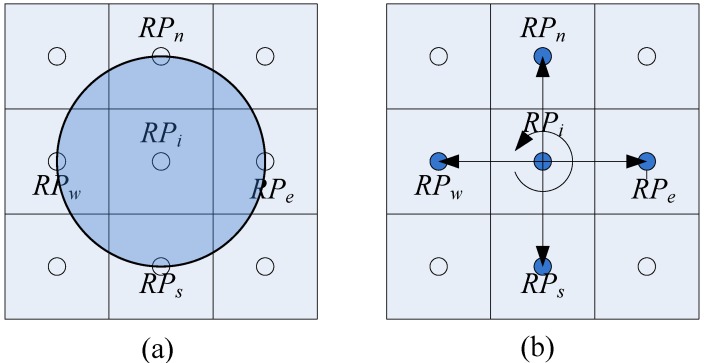
The pedestrian location transition to the next state: (**a**) the possible location after a sampling period; (**b**) consider the adjacent RPs as the possible pedestrian locations.

As mentioned before, the pedestrian locomotion is limited to a certain walking speed (2 m/s in this study) in an indoor environment. Figure 7a demonstrates that, after a sampling period (one second in this study), the next location can only be within a circle with a radius of two meters. If we collect the training sample at approximately the maximum speed and we set the RPs’ interval as equal to the maximum speed, the transition probability can be estimated through the FPR algorithm.

The main goal of the FPR algorithm is to consider the adjacent RPs as the possible pedestrian locations in the next state, as shown in Figure 7b. $V=\left\{v_{1},\;v_{2},\cdots ,v_{M}\right\}$ is the set of RSSI values along the planned path, and M is the number of the RPs along the path. It is obvious that $v_{i}$ is a RSSI sampling value at the position of ${\text{RP}}_{i}$. The transition probability from ${\text{RP}}_{i}$ to the adjacent RPs can be estimated as follows:

(1) Compare the $v_{i}$ and $v_{i\;+\;1}$ using the FPR arithmetic step two. The $O_{\left(i,i\;+\;1\right)}$ is the comparison result.
(2) The $O_{e},\;O_{w},\;O_{s},O_{n}$ are comparison results from ${\text{RP}}_{i}$ to adjacent RPs in the four directions. Because the radio map has been rebuilt, the $O_{e},\;O_{w},\;O_{s},O_{n}$ are already known. We can directly perform comparison operations between $O_{\left(i,i\;+\;1\right)}$ and $O_{e},\;O_{w},\;O_{s},O_{n}$ just as the FPR arithmetic steps 3 and 4.
(3) According to the comparison results between $O_{\left(i,i\;+\;1\right)}$ and $O_{e},\;O_{w},\;O_{s},O_{n}$, we can estimate the transition probability using a proportion method. To simplify the calculation we consider that the transition probability is in direct proportion to the logical XNOR operation result between $O_{\left(i,i\;+\;1\right)}$ and $O_{e},\;O_{w},\;O_{s},O_{n}$. No matter whether the RSSI is stronger or weaker, the more identical they are, the higher the transition probability is. The pseudo-code of this procedure is shown as follows:
  there are:
$O_{\left(i,i\;+\;1\right)}$, $O_{e},\;O_{w},\;O_{s},\;O_{n}$, $P_{c}$, $P_{e}$, $P_{w}$, $P_{s}$, $P_{n}$, $N_{c}$, $N_{e}$, $N_{w}$, $N_{s}$, $N_{n}$, $N_{\text{sum}}$;
${\text{RP}}_{e}$, ${\text{RP}}_{w}$, ${\text{RP}}_{s}$, ${\text{RP}}_{n}$, are adjacent RPs to ${\text{RP}}_{i}$ on four direction;
$O_{e}\;⊙\;O_{\left(i,i\;+\;1\right)}=\left(y_{1e},y_{2e},\cdots y_{\text{ne}}\right)$;
$O_{w}\;⊙\;O_{\left(i,i\;+\;1\right)}=\left(y_{1w},y_{2w},\cdots y_{\text{nw}}\right)$;
$O_{s}\;⊙\;O_{\left(i,i\;+\;1\right)}=\left(y_{1s},y_{2s},\cdots y_{\text{ns}}\right)$;
$O_{n}\;⊙\;O_{\left(i,i\;+\;1\right)}=\left(x_{1n},x_{2n},\cdots x_{\text{nn}}\right)$;
$N_{c}=\text{The number of zeros of}\;O_{\left(i,i\;+\;1\right)}$;
$N_{e}=\left(y_{1e}+y_{2e}+\cdots +y_{\text{ne}}\right)$;
$N_{w}=\left(y_{1w}+y_{2w}+\cdots +y_{\text{nw}}\right)$;
$N_{s}=\left(y_{1s}+y_{2s}+\cdots +y_{\text{ns}}\right)$;
$N_{n}=\left(y_{1n}+y_{2n}+\cdots +y_{\text{nn}}\right)$;
$N_{\text{sum}}=N_{c}+N_{e}+N_{w}+N_{s}+N_{n}$;
$P_{c}=P\left(L_{t\;+\;1}={\text{RP}}_{i}|L_{t}={\text{RP}}_{i}\right)=\frac{N_{c}}{N_{\text{sum}}}$
$P_{e}=P\left(L_{t\;+\;1}={\text{RP}}_{e}|L_{t}={\text{RP}}_{i}\right)=\frac{N_{e}}{N_{\text{sum}}}$
$P_{w}=P\left(L_{t\;+\;1}={\text{RP}}_{w}|L_{t}={\text{RP}}_{i}\right)=\frac{N_{w}}{N_{\text{sum}}}$
$P_{s}=P\left(L_{t\;+\;1}={\text{RP}}_{s}|L_{t}={\text{RP}}_{i}\right)=\frac{N_{s}}{N_{\text{sum}}}$
$P_{n}=P\left(L_{t\;+\;1}={\text{RP}}_{n}|L_{t}={\text{RP}}_{i}\right)=\frac{N_{n}}{N_{\text{sum}}}$
(4) If there are multiple training trajectories from other directions, we use the above steps to estimate the transition probability from other directions, and the final results are the arithmetic means of the transition probability from different directions. In this way, we use the FPR algorithm to estimate the transition probability, which provides strong support for the pedestrian positioning with the Viterbi algorithm.

## 5. Positioning Experiments and Results

To validate our algorithm and method, we perform some experiments in two real indoor environments. The first scenario is an office area located on the fourth floor of the YiFu building, and the second scenario is an open area located in the library.

To meet the needs of our experiment, the RSSI collection was performed using a Samsung S5 smartphone, in which the API supports scanning the RSSI with an interval of one second. If not otherwise specified, all measurements are collected with this smartphone. During the offline phase, we have taken a series of measures to improve the RSSI measurement accuracy. To mitigate the impact of the distribution difference of RSSI in different directions, we collected the measurements in four directions. Then, we collected 40 measurements at each reference point to decrease the random noise effects (thus 10 samples for each direction). Finally, we conducted the collection at midnight to avoid the interference caused by walking people. In the experiments, we mainly use the cumulative distribution function of error (CDF) and root mean square error (RSME) to calculate the positioning error.

### 5.1. Performance Evaluation in an Office Building

**Figure 8:**
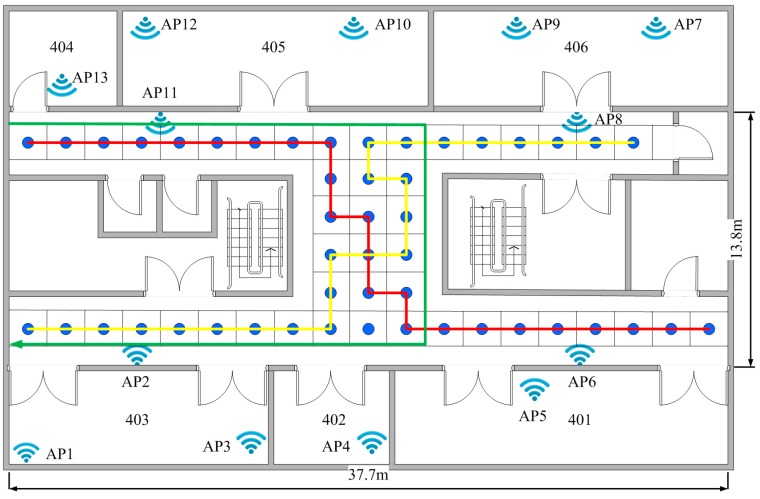
The layout of the fourth floor of the YiFu building.

This office area includes a horizontally H-shaped corridor and some rooms, as shown in Figure 8. For the purposes of simplifying the experimental procedure, we only chose the corridor area, which has a dimension of 37.7 m×13.8 m for localization. There are a total of 13 APs on this floor, four of them are RG-AP220-E APs which are deployed in the ceiling; the rest of the nine household APs are produced by different manufacturers, which are deployed in the different offices. The rough position of the APs is shown in Figure 8 as a wireless symbol. Due to the high-density deployment of the APs on the floor, almost all the localization area can receive 13 signals from the different APs. If there is an area where the signal cannot be received at that moment, it will be recorded as −99 dBm. The whole corridor area has been divided into 48 uniform 2 m×2 m grids, and the reference point is the center of each grid.

**Figure 9:**
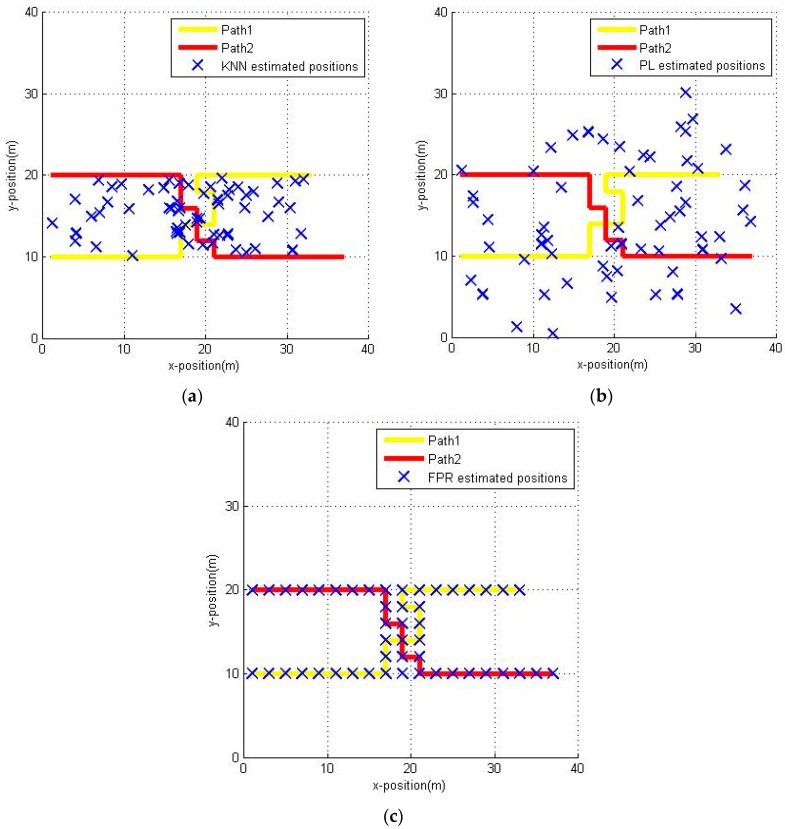
Estimated positions by different positioning algorithms: (**a**) KNN algorithm; (**b**) PL algorithm; (**c**) FPR algorithm.

After the radio map of this office area has been built, we can perform the experiments. The measurements are collected by a user walking alone the planned paths at about 1.5 m/s. The planned paths are shown in Figure 8 as a yellow line and red line, respectively. Both of them are 46 m long; that means we can collect 60 measurements while the user is walking at a speed of about 1.5 m/s. We estimated the user position by three methods, namely the KNN algorithm (k=8), the probabilistic location (PL) [19,20] algorithm and the fuzzy pattern recognition algorithm, respectively. The estimated positions of the user are shown in Figure 9.

From Figure 9, we can clearly see that because of the RSSI deviations caused by the different measuring conditions (static or dynamic), the estimated positions present a cloud-like pattern in Figure 9a,b. That means there are lots of ambiguities in the results when we use the KNN algorithm and PL algorithm. Unlike the two left figures, Figure 9c shows that the estimated positions are punctuated clearly and accurately at the reference points along the planned path. That is because the path just passes through the RPs, and the FPR algorithm selects the adjacent RPs as the candidate pedestrian position. Actually, the performance of the FPR algorithm is better than that of the other algorithms but it is not as strong as Figure 9 shows.

**Figure 10:**
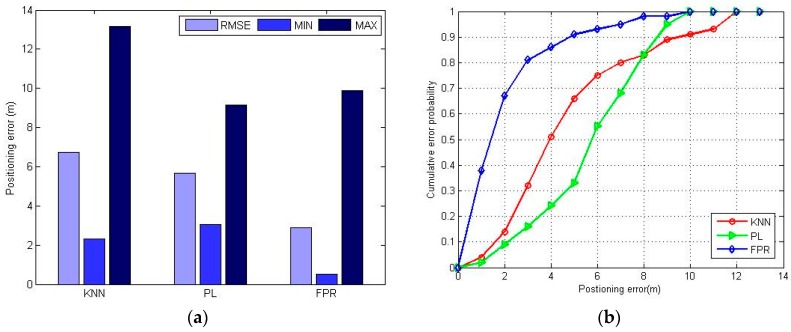
The positioning error of KNN, PL and FPR algorithm: (**a**) maximum positioning error, minimum positioning error and RMSE of different positioning methods; (**b**) CDF of different positioning methods.

To fairly compare the positioning error, Figure 10 shows the experimental results of RMSE, minimum error, maximum error and cumulative error probability of the different positioning algorithms. Obviously, the FPR algorithm performs better than the KNN and PL algorithm. The RMSE and the minimum error of the FPR algorithm are both minimal, but the maximum error of the FPR algorithm is greater than that of the PL algorithm. This is because the FPR algorithm introduces an accumulating error with each positioning process when direction-hopping happens. The direction-hopping means the ambiguities of the positioning result are too numerous to distinguish the direction of the next position.

**Figure 11:**
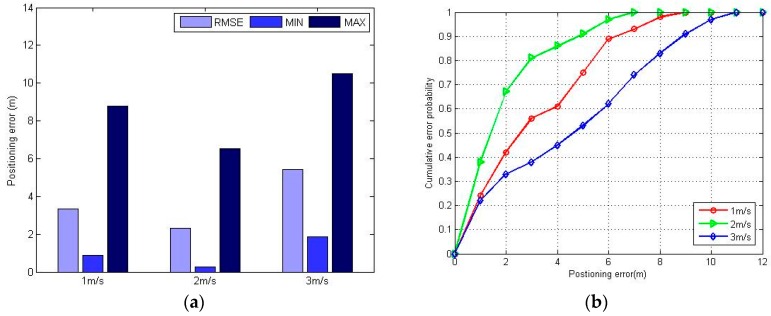
The positioning error at different walking speeds using FPR algorithm: (**a**) maximum positioning error, minimum positioning error and RMSE at different speed; (**b**) CDF at different speed.

Figure 11 shows the experimental results of the positioning error using the FPR algorithm when the pedestrian walks at different speeds. In the case where the pedestrian speed is no more than 2 m/s, the dynamic positioning error only fluctuates slightly at different speeds, and the positioning error is smaller when the speed is approaching 2 m/s. When the pedestrian speed is 3 m/s, the positioning error becomes larger. The main factor is that when the distance a pedestrian walks in a second is more than the RPs’ interval—and the distance a pedestrian moves may span one or two grid areas—the FPR algorithm has difficulties to distinguish the real position because it only considers the RSSI variation trend, and this ambiguity will also accumulate in each positioning process and finally bring about a large positioning error.

**Figure 12:**
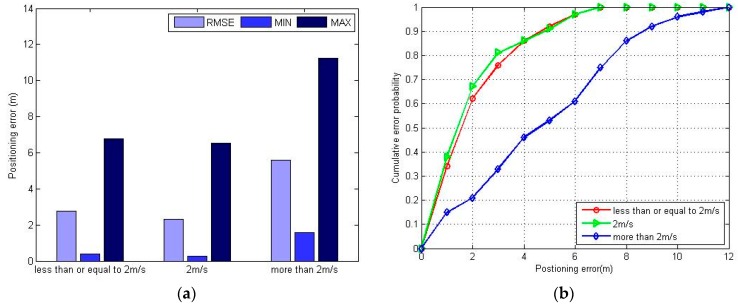
The positioning error under uniform and variable speed using FPR algorithm: (**a**) maximum positioning error, minimum positioning error and RMSE at uniform and variable speeds; (**b**) CDF at uniform and variable speeds.

Figure 12 shows the positioning error of the proposed FPR algorithm at uniform and variable speeds. When the variable speed range is less than or equal to 2 m/s, the positioning error is slightly worse than at the uniform speed of 2 m/s. Otherwise, the positioning accuracy will decrease rapidly. From Figure 11 and Figure 12, we know that FPR algorithm offers better accuracy when the pedestrian walking speed is no more than the RPs’ interval per second (here is 2 m/s), either at uniform or variable walking speed. The initial position of each estimation is replaced iteratively by adjacent RPs in the FPR algorithm, which can easily compensate for errors of less than the RP interval, but it cannot decrease the error accumulation caused by walking too fast.

**Figure 13:**
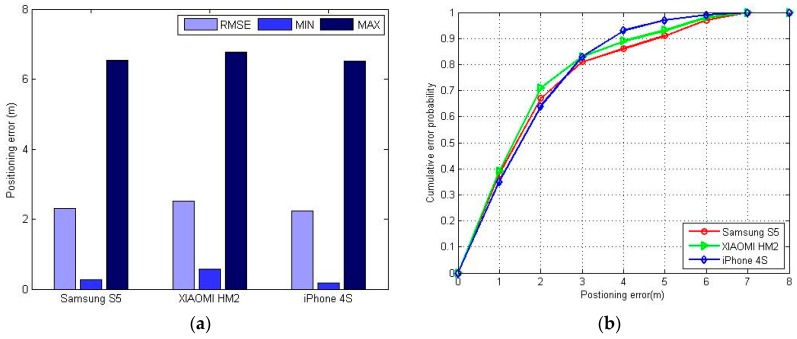
The positioning error with different smartphones using the FPR algorithm: (**a**) maximum positioning error, minimum positioning error and RMSE with different devices; (**b**) CDF with different devices.

To test the adaptability of the FPR algorithm to different devices, we used a XIAOMI HM2 and an iPhone 4S (IOS 6.1.3, Apple, Cupertino, CA, USA) to repeat the experiments. There was little change in the positioning error, which means the FPR algorithm has good adaptability to different smartphones, as can be seen in Figure 13.

From the above experimental results, we can see that the FPR algorithm can offer better positioning accuracy over a certain range of speeds and has good adaptability to different devices, but it also has two drawbacks: one is that its positioning accuracy is limited by the RP interval, the other is the accumulating positioning error when direction-hopping is happening.

In the HMM method testing, the red line and the yellow line are treated as the model training trajectories, and the walking path is the green line as shown in Figure 8. We use the FPR algorithm to estimate the transition probability with the measurements collected at the speed of 2 m/s from two directions, one time from east to west, and the other time from west to east. To collect the measurements for HMM method testing, a user walks at a variable speed which is no more than 2 m/s with the Samsung S5 (Samsung, Tianjin, China) following the green line from the door of the room marked as 404 to the west door of room 403. For comparison, we also use the method mentioned by Liu et al. [10] to estimate the pedestrian position. The method HMM.acc combines the HMM with the accelerometer sensor to compute the pedestrian position. The HMM.noacc is an alternative solution if the accelerometer sensor is unavailable, which needs to assume the pedestrian walks at a uniform speed.

**Figure 14:**
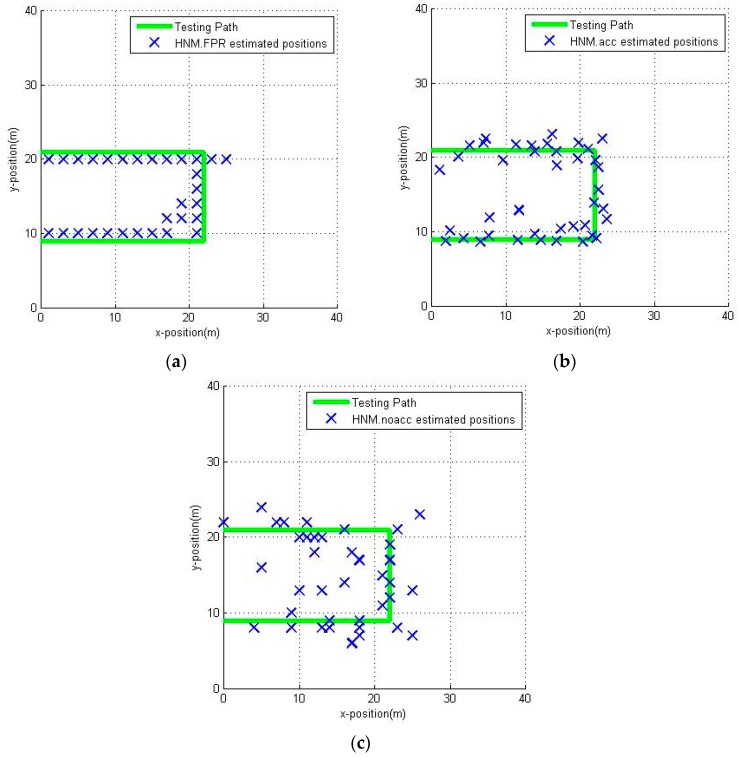
Estimated positions by different positioning methods: (**a**) HMM.FPR; (**b**) HMM.acc; (**c**) HMM.noacc.

**Figure 15:**
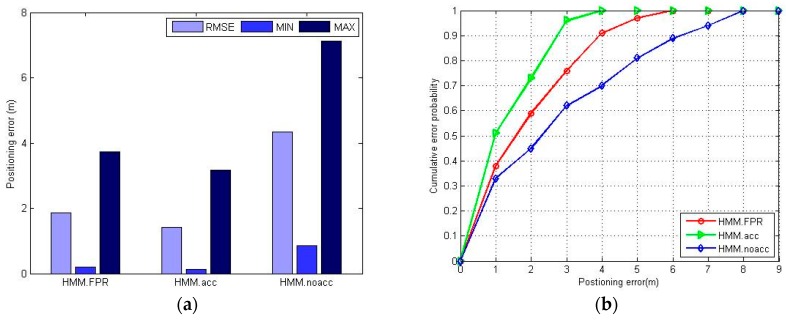
The positioning errors of HMM.FPR, HMM.acc, HMM.noacc: (**a**) maximum positioning error, minimum positioning error and RMSE of different positioning methods; (**b**) CDF of different positioning methods.

The estimation of the user position along the green path is shown in Figure 14. Because of the characteristics of the FPR algorithm, all the estimated positions are on the RPs, thus reducing the positioning accuracy to a certain extent but within the range of acceptability. Also, from Figure 14 we can learn that, because of the incomplete coverage of the planned path, the estimated position has been hopped in the lower-middle area of the corridor. The HMM.acc has the best positioning accuracy, as the estimated positions with HMM.acc are closer to the walked path in Figure 14b. It seems like the HMM.noacc has the worst positioning accuracy since some estimated positions cluster together in Figure 14c. The results are further compared in Figure 15.

It can be seen in Figure 15 that the HMM.FPR (Fuzzy Pattern Recognition) indeed has better positioning accuracy than HMM.noacc, but slightly worse than HMM.acc. The HMM.acc utilizes an accelerometer sensor to provide the pedestrian dynamics information, which is useful to improve the positioning accuracy. The HMM.noacc offers the worst positioning accuracy because the pedestrian walks at variable speeds, and the assumption of uniform speed will compromise the positioning accuracy. HMM.FPR uses the estimated transition probability to mitigate the effect of direction-hopping, which will effectively reduce the maximum positioning error.

### 5.2. Performance Evaluation in a Library Building

**Figure 16:**
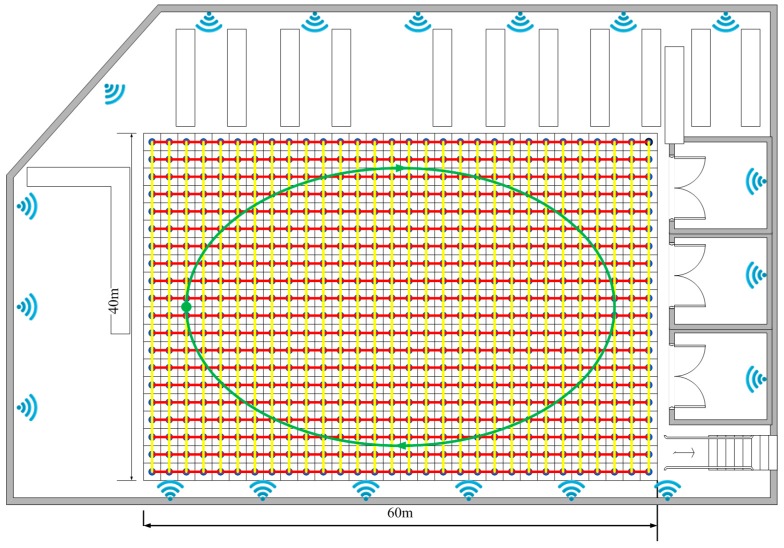
The layout of the first floor of the university library.

After testing the method in the office area, we chose a relatively open indoor area to further evaluate our method. The open area is on the first floor of our university library (Beijing, China), which has dimensions of 60 m×40 m. There are a total of 19 APs covering this area, all of which are RG-AP220-E APs deployed in the ceiling. Because there are not so many barriers, some APs can reach this area even at a great distance. The rough position of the APs is given in Figure 16. The whole area has been divided into 600 uniform 2 m×2 m grids, and the RP interval is 2 m.

As in Figure 8, we use three colors to present the planned path and the testing path. The red line and the yellow line are the planned path for training the transition probability. In this testing, we collect training samples from four directions to maximize precision. The green line is the only testing path that means all methods use the same measurements to estimate pedestrian position. The starting point is marked as a green dot and arrows are used to mark the walking direction. The 100 test measurements are collected by a user walking along the green path at a variable speed of less than 2 m/s when the library is open.

**Figure 17:**
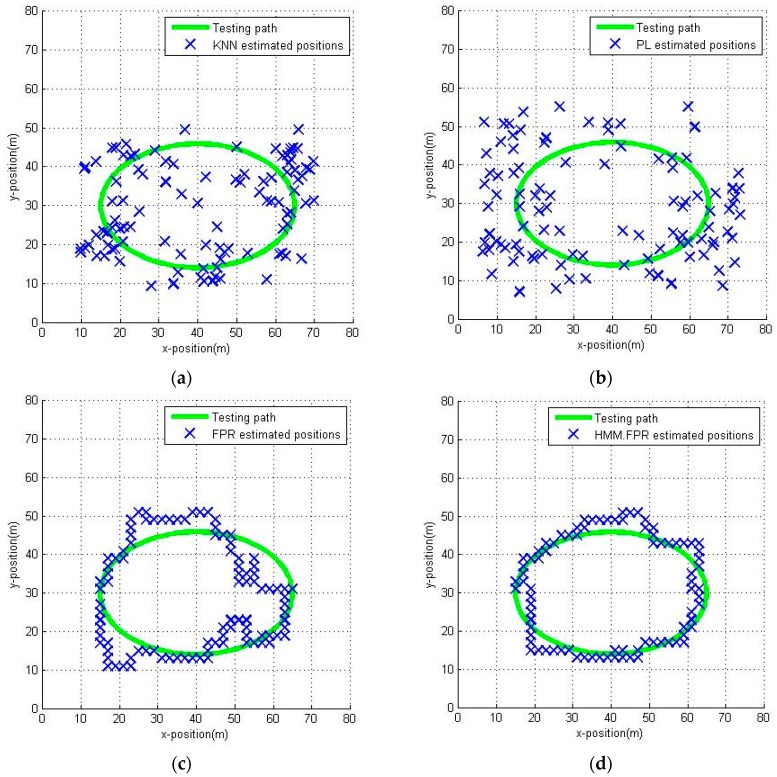

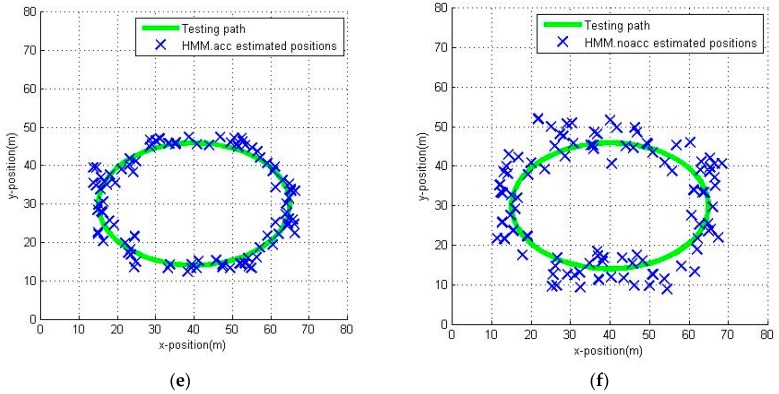
Estimated positions by different positioning methods in the open area: (**a**) KNN; (**b**) PL; (**c**) FPR; (**d**) HMM.FPR; (**e**) HMM.acc; (**f**) HMM.noacc.

**Figure 18:**
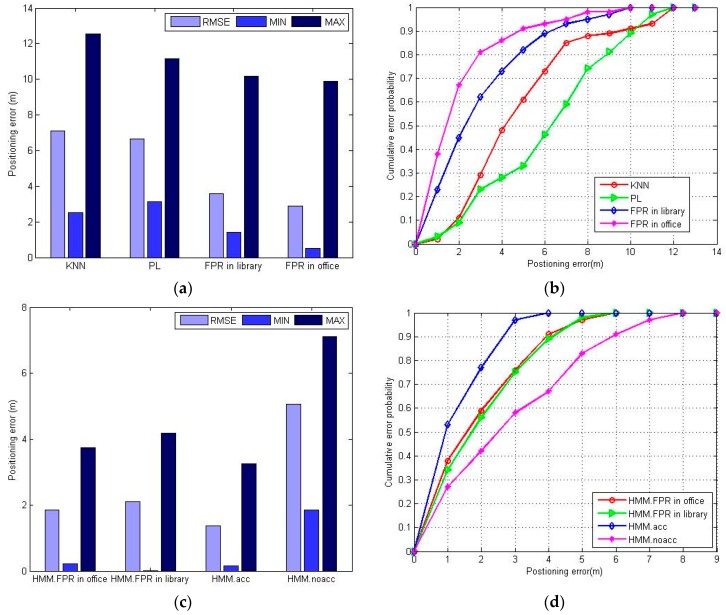
The positioning error of the different algorithms and methods: (**a**) maximum positioning error, minimum positioning error and RMSE of different positioning algorithms; (**b**) CDF of different positioning algorithms; (**c**) maximum positioning error, minimum positioning error and RMSE of different positioning methods; (**d**) CDF of different positioning methods.

The positions in the open area estimated by different methods are shown in Figure 17. The KNN and the PL algorithm have the worst positioning accuracy because of the numerous ambiguous results shown in Figure 17a,b. The FPR algorithm is much better than the KNN and PL algorithm but also has some estimated positions with a large distance to the test path. The errors caused by direction-hopping are greater in an open area. The HMM.FPR performs better than HMM.noacc but worse than HMM.acc, and there are few large distance errors in Figure 17d, indicating that the HMM plays an important role in mitigating the effect of direction-hopping. The HMM.acc also has the best positioning accuracy, and the open area seems to have no influence on HMM.acc and HMM.noacc. The RMSE and the CDF of the different methods are shown in Figure 18.

It is more obvious in Figure 18a that the FPR algorithm performs better than the KNN and PL algorithms. To compare the influence of the different scenarios, we give the RMSE (Root Mean Square Error) and the CDF (Cumulative Distribution Function) of the FPR algorithm in the office scenario in Figure 18a,b. Although the FPR algorithm shows a slight performance degradation in the open area, the RMSE of this algorithm is still less than 4 m, and more than 70% of the positioning errors are no more than 4 m as shown in Figure 18b. The influence of the different scenarios indicate that the FPR algorithm has better positioning accuracy in the space-constrained area.

In Figure 18c,d. we also give the error results of the HMM.FPR method in the office. Unlike the FPR algorithm, the HMM.FPR works well in both scenarios. The RMSE of the HMM.FPR is about 2 m and the accuracy of the HMM.FPR within 3 m is about 75%, which is acceptable for pedestrians in an indoor area. Also, this method mitigates the effect of the direction-hopping of the KNN algorithm in the open area. As expected, the HMM.FPR has better positioning accuracy than HMM.noacc, but one that is worse than HMM.acc. The experimental results show that the proposed HMM.FPR method can obtain satisfactory accuracy in pedestrian dynamic positioning without using other sensors.

## 6. Conclusions

In this paper. A method for precise pedestrian positioning in an indoor environment using a trained HMM with a fuzzy pattern recognition algorithm was proposed. The presented fuzzy pattern recognition algorithm can offer better positioning accuracy during pedestrian walking because this algorithm utilizes the RSSI variation trend of the different locations to replace the specific RSSI values, which display significant deviations during dynamic measurement. To improve the positioning accuracy and mitigate the effect of direction-hopping, we introduce the trained HMM with the FPR algorithm in the WLAN fingerprint system. We use a rebuilt radio map and training trajectories to train the HMM with the FPR algorithm; thus, the most likely position of the pedestrian can be estimated by employing the Viterbi algorithm in the HMM. In our experiments, we demonstrate that the fuzzy pattern recognition algorithm has acceptable positioning accuracy and good environmental adaptability, and the accuracy is better after we embedded the fuzzy pattern recognition algorithm into the trained HMM. Future studies will focus on optimizing features of the proposed method, such as the AP selection, planned path selection and multi-sensor fusion.
